# Translation and validation of Chinese version of sense of competence in dementia care staff scale in healthcare providers: a cross-sectional study

**DOI:** 10.1186/s12912-022-00815-3

**Published:** 2022-01-30

**Authors:** Yayi Zhao, Li Liu, Yaping Ding, Ye Shan, Helen Y. L. Chan

**Affiliations:** 1 School of Nursing, Nanjing University of Chinese Medicine, No. 138 Xianlin Avenue, Xianlin District, Nanjing, 210000 Jiangsu Province China; 2 Xiangya Nursing School, Central South University, Changsha, Hunan Province China; 3 School of Nursing, Nanjing Medical University, Nanjing, Jiangsu Province China; 4 Nursing Department, Suzhou High-tech Zone Yangshan Street Community Health Service Center, Suzhou, China; 5 The Nethersole School of Nursing, Faculty of Medicine, The Chinese University of Hong Kong, Hong Kong, China

**Keywords:** Chinese, Dementia, Reliability, Sense of competence, Validity

## Abstract

**Background:**

Healthcare providers’ dementia-care competence is crucial for quality dementia care. A reliable and valid instrument is needed to assess the gaps in their dementia-care competence, and thereby identifying their educational needs. Therefore, this study aims to translate the 17-item Sense of Competence in Dementia Care Staff (SCIDS) scale into Chinese (SCIDS-C) and to validate the SCIDS-C among Chinese healthcare providers.

**Methods:**

The translation procedure followed the modified Brislin’s translation model. A cross-sectional survey was conducted using the translated version. The validity, including content validity, confirmatory factor analysis, concurrent validity and known-groups validity, was tested. Reliability in terms of internal consistency and test-retest reliability with a 2-week interval was evaluated.

**Results:**

A total of 290 healthcare providers in 12 nursing homes and a hospital completed the survey. The scale-level content validity index was .99. The confirmatory factor analysis model marginally supported the original 4-factor structure. Positive but weak correlations were noted between the total score of the SCIDS-C and that of the Dementia Knowledge Assessment Scale (r = .17, *p* = .005) and Approaches to Dementia Questionnaire (r = .22, *p* < .001), suggesting acceptable concurrent validity. Differences between health professionals and care assistants were significant in two subscales scores. The internal consistency of the scale was high, with Cronbach’s α of .87. Test-retest reliability was demonstrated with intra-class correlation coefficient of 0.88.

**Conclusions:**

The SCIDS-C demonstrated acceptable reliability and validity although the known-groups validity between health professionals and care assistants was not fully established. It can be used to measure the level of sense of competence and as an outcome measure in educational intervention aiming at improving dementia care among Chinese healthcare providers.

**Supplementary Information:**

The online version contains supplementary material available at 10.1186/s12912-022-00815-3.

## Background

Dementia is now recognised as a global public health priority [1]. Over 55 million people are living with dementia worldwide [2]. In China, it is estimated that 15.07 million people aged above 60 years old are living with dementia, accounting for approximately 6.0% of the older population [3]. China is one of the countries with the fastest growing population of people with dementia because of its ageing population [4, 5].

Dementia is a complex condition associated with an increased risk of hospitalisation and long-term care. Meta-analysis showed that the relative risk of hospital admission among older adults with dementia was 1.42 as compared to those without, after adjusting for age, sex and comorbidity, and the risk was independent of the dementia severity [6]. As the disease progress, nursing homes are always the last place of care when families cannot provide care for their relatives with dementia. Therefore, dementia care competence among healthcare providers in these care settings is crucial because it determines the quality of care provided to this particular group [7–9].

Competence is the ability to apply the discipline-related skills and knowledge in professional practice with sound judgement, develop positive interpersonal relationships and evaluate practice outcomes (e.g., quality of care) by standards [7]. Staff’s incompetence in dementia care is associated with task-oriented care approaches [10, 11] and use of inappropriate care management, such as the use of physical or chemical restraint [8, 12]. By contrast, person-centred care provided by staff, including staying with residents during care, tailoring the pace of care to residents and focusing on the person beyond the task, are linked to residents’ positive mood, such as pleasure and interest during interpersonal contact [13]. Gerritsen et al. demonstrated the positive relationships between staff’s hopeful attitude towards people with dementia and residents’ social well-being and decreased challenging behaviours [8].

Preparedness of healthcare providers for dementia care in China is inadequate [14–17]. Literature generally showed that healthcare providers, including healthcare professionals and care assistants, in nursing homes and hospitals lack relevant caregiving knowledge and skills [18, 19]. Some nursing homes do not accept admission application for people with dementia and discharge residents who develop dementia during their stay because of inadequate staff competence and service to meet the care needs [20]. Healthcare providers also express concern about huge caregiving burden and high level of work strain due to difficulties in managing challenging behaviours exhibited by people with dementia [16, 21].

A reliable and valid instrument that can identify their educational needs that influence their perceived competence in dementia care is the crucial. Sense of competence in dementia care referred to self-perceived competence or confidence in addressing the care need of people with dementia [22, 23]. Higher sense of competence is associated with caregivers’ positive outcomes, such as positive appraisal of caring and gratitude [24], greater job satisfaction, positive attitudes towards people with dementia and less caregiver burden [22, 25, 26].

Three instruments were developed to assess healthcare providers’ sense of competence in dementia. Sense of Competence Questionnaire and its corresponding short form have satisfactory psychometric properties among family caregivers of people with dementia [27, 28]. Confidence in Dementia Scale is developed to assess hospital staff’s confidence in working with people with dementia, with good reliability and validity [29, 30]. Sense of Competence in Dementia Care Staff (SCIDS) scale developed specifically for healthcare providers involved in dementia care [22]. Among these three instruments, SCIDS has been proved to be psychometrically sound and widely used in assessment and evaluation of healthcare training programmes across various disciplines and care settings [9, 23, 31, 32]. To the best of our knowledge, no instrument is validated for evaluating sense of competence in dementia care in Chinese communities.

## Methods

### Aim

The aim of this study was to translate the 17-item SCIDS from English to simplified Chinese and to examine its reliability and validity among healthcare providers in nursing homes and hospitals in China.

### Design

Psychometric properties of the SCIDS-C were validated through a cross-sectional study conducted between June and August 2019 in Jiangsu Province, mainland China. ﻿This study was reported under the guideline of Strengthening the Reporting of Observational Studies in Epidemiology checklist for observational research [33] (Supplementary file 1).

### Participants and setting

Participants were healthcare providers recruited from 12 nursing homes and three departments in a general hospital in Nanjing, Jiangsu Province. The inclusion criteria were healthcare providers who (a) were engaged in care for patients or residents, such as care assistants, nurses, doctors, physiotherapists, and social workers, (b) could read simplified Chinese, and (c) were willing to complete the questionnaires. We excluded those who were not engaged in patient care, such as administrative staff. A rule of thumb recommended that at least 10 respondents were necessary for each item for factor analysis [34]. Because SCIDS has 17 items, at least 170 participants were needed for this study. Participants’ demographic characteristics, including age, sex, education level, workplace, profession, and clinical experience, were collected.

### Instruments

#### SCIDS

SCIDS is to assess the sense of competence in dementia care specific to care staff of people with dementia. The 17 items are categorized into four subscales, ‘Professionalism’ (five items), ‘Building Relationships’ (four items), ‘Care Challenges’ (four items) and ‘Sustaining Personhood’ (four items). The items were rated on a 4-point Likert scale, 1 = ‘*not at all*’, 2 = *‘a little bit*’, 3 = ‘*quite a lot*’ and 4 = ‘*very much*’. The total score is the sum of all item scores, ranging from 17 to 68. Higher scores indicate higher level of sense of competence in dementia care. The English version showed good internal consistency with the Cronbach’s α of .91 for the full scale [22].

### Dementia knowledge assessment scale (DKAS)

The 25-item DKAS which assesses dementia knowledge [35] was used to test the concurrent validity of SCIDS-C because dementia knowledge towards people with dementia was used to confirm the concurrent validity of the English version of SCIDS [22]. For the DKAS, there are five responses to each item, namely ‘*false’, ‘probably false’, ‘probably true’, ‘true’ and ‘I don’t know’*. Two points are scored by true statement with true response and false statement with false response. One point is scored by the response of ‘*probably true*’ to a true statement or ‘*probably false*’ to a false statement. No point is scored by the wrong responses or ‘*I don’t know*’. The total score is the sum of the points of all items, with a maximum of 50. DKAS has been translated and validated among Chinese healthcare providers with Cronbach’s α of .77, indicating an acceptable internal consistency [17]. We hypothesised that the scores of the DKAS would be positively associated with the scores of the SCIDS-C.

### Approaches to dementia questionnaire (ADQ)

ADQ which measures care staff’s attitudes towards people with dementia was also used to test the concurrent validity of SCIDS-C [36]. It includes 12 items on two factors: ‘Hope’ (five items) and ‘Person-centred’ (seven items). The responses are 5-point Likert, from ‘*strongly agree*’ to ‘*strongly disagr*ee’. For the five negatively phrased items, the scores are reversed. The total score is 12–60 with higher scores indicating more positive attitudes. The Chinese version showed good internal consistency (Cronbach’s α = .74) among healthcare providers in China. We hypothesised that the scores of the ADQ and the SCIDS-C would be positively associated.

### Translation

The translation procedure followed the modified Brislin’s translation model [37]. Firstly, in the forward translation, two bilingual researchers, with postgraduate qualifications and specialized in geriatric nursing, translated the English version into simplified Chinese. Secondly, back translation was performed by two other bilingual translators, who were experienced nurses and had no idea about the original English version, translated the Chinese version into English. Thirdly, agreement on the expression of the simplified Chinese version of the SCIDS (SCIDS-C) was reached through a meeting with all translators and a researcher (first author) in dementia care. Finally, to assess the semantic equivalence, the SCIDS-C and the back translated English version were checked by an additional bilingual nursing researcher in dementia with oversea education background and postgraduate qualification in geriatric nursing.

### Data collection

The first author introduced the study to the potential respondents with an information sheet during staff meeting. A questionnaire, including the DKAS, SCIDS-C, ADQ and demographic characteristics, was distributed to those who were interested to participate in the study. The participants were asked to self-administer the questionnaires independently and returned the completed questionnaires to the investigator directly within the day. It took approximately 15–20 min to finish the questionnaire. A convenient sample of 56 participants were invited to complete the SCIDS again 2 weeks later for examining its test-retest reliability. The 2-week interval was suggested because it was long enough for participants to forget their previous answers [38].

### Ethical considerations

Permission for translating the SCIDS into simplified Chinese was obtained from the original author. Approval for conducting this study was obtained from the ethical committee of Survey and Behavioural Research Ethics, The Chinese University of Hong Kong (NO. SBRE-18-557) and management levels of the involved nursing homes and hospital. The Declaration of Helsinki was adhered to during this study. Voluntary and anonymous participation without any incentives were emphasized. The participants were assured to have the right to withdraw from the study without any negative effects on them. Data were being kept confidential and for research purpose only.

### Statistical analysis

Data were analysed by using SPSS (version 25.0) for Windows (IBM, Armonk, NY, USA). The data was described by using mean and SD for continuous variables with normal distribution, median and interquartile for continuous variables with skewed distribution, and frequency and percentage for categorical variables (e.g., gender, education level, workplace and professions). Normal distribution was tested by using skewness and kurtosis values, which within − 2 to 2 are normal [39].

Content validity of the SCIDS-C was appraised by an expert panel in dementia care in mainland China. The six experts included a senior nurse and two physicians working in a teaching hospital, a social worker working in a nursing home and two university academics involved in aged care research. The experts’ mean clinical experience in dementia was 17.7 years. They were invited to rate the relevance of each item on a 4-point Likert scale (from 1 = not relevant to 4 = highly relevant). The content validity index of each item (I-CVI) was calculated based on the formula: the total number of answers being rated 3 or 4 divided by the total number of answer [40]. The scale-level CVI (S-CVI) was the mean of all I-CVI. The criteria for good content validity were I-CVI ≥ .78 and S-CVI ≥ .90 [41].

The SCIDS-C was then distributed to 10 healthcare providers, including nurses, doctors and care assistants working in a nursing home and a hospital, to test the face validity. They were asked to appraise and comment on the clarity and ease to understand each item through face-to-face interviews. Their comments and suggestions were recorded in writing immediately during the interviews for the research team reference. The team then discussed and revised the wordings to enhance the comprehensibility, as necessary.

Confirmatory factor analysis (CFA) was conducted to confirm the replicability of the original 4-factor structure of SCIDS in this study [22]. Before CFA, the Kaiser-Meyer-Olkin Measure of Sampling Adequacy and Bartlett’s Test of Sphericity were calculated to assess the appropriateness of the data. CFA of SCIDS-C was performed by using a robust maximum likelihood method which allows normality assumption for the scale item is slightly or moderately violated in AMOS version 24.0. The comparative fit index (CFI), Tucker–Lewis index (TLI), root mean squared error of approximation (RMSEA) and standardized root mean square residual (SRMR) were employed to evaluate the goodness-of-fit of the CFA model. The criteria of a good model fit were CFI and TLI values ≥ .95, RMSEA < .06 and SRMR < .10 [42]. Pearson correlation coefficients among subscales were calculated to examine the degree of overlapping and redundancy.

Concurrent validity of the SCIDS-C was established by examining the correlation between the scores of SCIDS-C and DKAS, and the scores of SCIDS-C and ADQ using Pearson correlation coefficients. DKAS and ADQ were selected for the following three reasons. Firstly, there was no Chinese validated instrument available to measure healthcare providers’ sense of competence in dementia care. Secondly, studies have found positive association among dementia knowledge, attitudes and sense of competence [43, 44]. Thirdly, DKAS and ADQ has been validated among Chinese healthcare providers [17].

Known-groups validity was evaluated by comparing the mean scores of SCIDS-C between health professionals and care assistants using independent t-test. It was hypothesised that care assistants would have lower SCIDS-C scores than healthcare professionals because they generally received less training in dementia care.

The reliability was determined based on internal consistency and test-retest reliability. Cronbach’s α coefficient was used to evaluate the internal consistency of the SCIDS-C and the subscales. Average inter-item correlation for subscales was calculated to ensure that the items were measuring the same construct but with sufficient uniqueness [45, 46] The criterion of acceptable internal consistency was Cronbach’s α of > .70 [47]. Average inter-item correlation coefficients ranging from .15 to .50 were considered acceptable [45]. The intra-class correlation coefficient (ICC) was calculated to evaluate the test-retest reliability of the SCIDS-C with value > .70 indicating acceptable reliability [40].

## Results

### Participants’ characteristics

A total of 290 completed questionnaires for the cross-sectional study were included in the analysis (Table 1). The respondents’ mean age was 37.2 years (SD = 12.1), with a median clinical experience of 6 years. Female participants accounted for 76.6%. More than half of the participants had education level at junior college or below (59.4%). Two-thirds were currently working in nursing homes (64.5%). Staff of professional ranks accounted for 70.7% among all participants.

**Table 1:** Participants’ demographic characteristics (*N* = 290)

Characteristics		***n*** (%) ^a^
Age, (mean ± SD)		37.2 ± 12.1 (range: 20–67)
Clinical experience (year) ^b^, median (interquartile)		6 (2,10) (range: 0–35)
Sex	Male	66 (22.8)
	Female	222 (76.6)
Education level	Junior high school or below	75 (25.9)
	Junior college	97 (33.5)
	College degree and above	117 (40.3)
Workplace	Tertiary hospitals	70 (24.1)
	Other hospitals	19 (6.6)
	Nursing homes	187 (64.5)
Profession	Care assistants	82 (28.3)
	Health professionals	205 (70.7)

*Note.* ^a^ frequency (percentage), unless specified; ^b^ skewed distributed (Skewness value = 1.992, Kurtosis value = 4.838)

### Content and face validity

The I-CVI of the SCIDS-C ranged from .83 to 1.0 and the S-CVI was .99, suggesting acceptable content validity. No changes in wording of SCIDS-C were made because the expert panel or participants in the face validity process did not have any concerns about the clarify of expressions.

### CFA

**Fig. 1:**
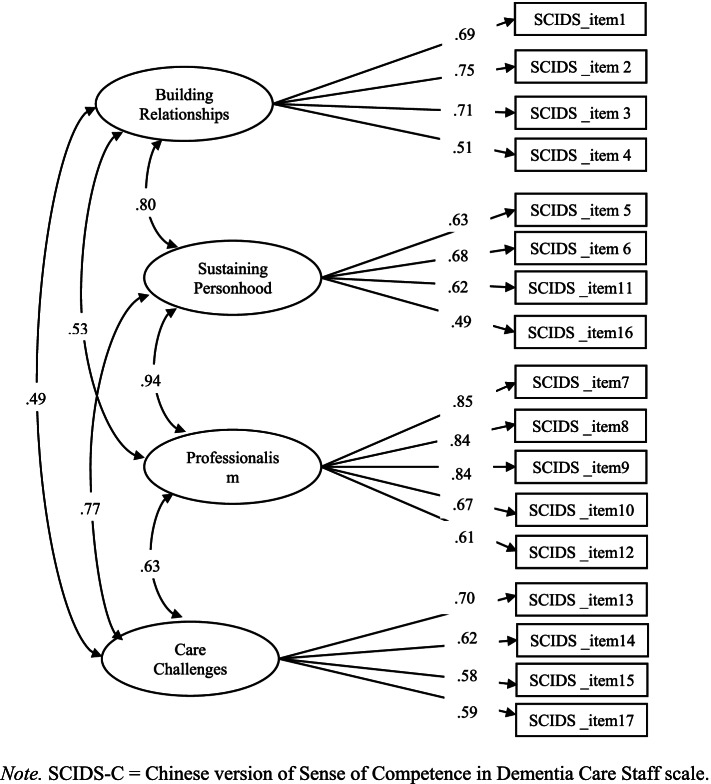
Confirmatory factor analysis of the 4-factor structure of the SCIDS-C

Factorial validity of the SCIDS-C was established based on the original 4-factor structure of the SCIDS. The Kaiser-Meyer-Olkin Measure of Sampling Adequacy (.860) and Bartlett’s Test of Sphericity (χ^2^ = 1775.97, *p* < .001) indicated that the data were appropriate for factor analysis. The CFA model (Fig. 1) based on the data from the current study was marginally fit (χ^2^ = 310.78, df = 118, *p* < .001; CFI = .96; TLI = 0.95; RMSEA = .079; SRMR = .099). The factor loadings of all items were ≥ .40 and were significantly loaded onto the corresponding factor. The correlations among the subscale scores ranged from .29 to .65 (Table 2), suggesting that they were assessing different domains.

**Table 2:** Differences between care assistants and health professionals in SCIDS-C scores

	Total score	Care assistants (Mean ± SD)	Health professionals (Mean ± SD)	t	*p*
Professionalism	14.8 ± 2.7	15.9 ± 2.7	14.4 ± 2.6	4.255	<.001
Building Relationships	10.3 ± 2.1	9.9 ± 1.8	10.5 ± 2.2	−2.189	.030
Care Challenges	10.5 ± 2.0	10.5 ± 1.8	10.5 ± 2.1	0.316	.752
Sustaining Personhood	11.4 ± 1.9	11.6 ± 1.8	11.4 ± 2.0	0.900	.370
Total SCIDS score	46.9 ± 7.0	47.9 ± 5.8	46.6 ± 7.3	1.516	.120

*Note.* SCIDS-C = Chinese version of Sense of Competence in Dementia Care Staff scale

### Concurrent validity

There was a positive but weak correlation between the total scores of the SCIDS-C and the DKAS (r = .17, *p* = .005) and between the total scores of the SCIDS-C and the ADQ (r = .22, *p* < .001). Two out of four subscale scores, ‘Building Relationships’ and ‘Sustaining Personhood’ of the SCIDS-C, were positively correlated with the total score of the DKAS (r = .29 and .19, respectively, *p* < .01) and the total score of the ADQ (r = .32 and .23, respectively, *p* < .001). The scores of the other two subscales, ‘Professionalism’ and ‘Care Challenges’, were not significantly associated with the total scores of DKAS (r = .03 and .05, respectively, *p* > .05) or ADQ (r = .09 and .08, respectively, *p* > .05).

### Known-groups validity

Differences in the total SCIDS-C scores between health professionals (46.6 ± 7.3) and care assistants (47.9 ± 5.8) were not statistically significant (*p* = .120). Significant differences between these two groups were detected in the subscale scores of ‘Professionalism’ (*p* < .001) and ‘Building Relationships’ (*p* = .030), but not for the subscales ‘Care Challenges’ and ‘Sustaining Personhood’ (*p* = .752 and .370, respectively) (Table 2).

### Internal consistency

The Cronbach’s α of the SCIDS-C was .87, indicating satisfactory internal consistency of total scale. The Cronbach’s α of the four subscales were .82 (Professionalism), .69 (Building Relationships), .66 (Care Challenges) and .61 (Sustaining Personhood). The average inter-item correlations of the four subscales were .48 (Professionalism), .36 (Building Relationships), .33 (Care challenges) and .28 (Sustaining Personhood). All the correlations were within an acceptable range from .15 to .50, suggesting that the items were correlated but unique.

### Test–retest reliability

The ICC of the SCIDS-C and the four subscales at the 2-week interval were .88 (overall), .85 (Professionalism), .86 (Building Relationships), .86 (Care Challenges), and .83 (Sustaining Personhood), suggesting satisfactory test-retest reliability.

### SCIDS-C scores

The mean total score of the SCIDS-C of the respondents in this study was 46.9 (SD = 7.0) out of 68, ranging from 28 to 67. No ceiling or floor effects were observed because the skewness and kurtosis values were 0.242 and 0.457, respectively. The three items with the highest scores were: item 7 ‘*keep up a positive attitude towards the people you care for’*, item 8 *‘keep up a positive attitude towards the relatives of a person with dementia’* and item 11 *‘protect the dignity of a person with dementia in your work’*. They scored the lowest in four items: item 4 ‘*balance the needs of the person with dementia with their relative’s wishes and the service’s limitations’*, item 15 ‘*offer stimulation (for the mind, the senses and the body) to a person with dementia in your daily work*’, item 2 ‘*understand the way a person with dementia interacts with the people and things around them*’ and item 3 ‘*engage a person with dementia in a conversation’*. Mean scores and frequency of responses to each item in the SCIDS-C were shown in Supplementary file 2.

## Discussion

This study translated the SCIDS from English to simplified Chinese and examined the psychometric properties of the translated version among healthcare providers in nursing homes and hospital in mainland China. The construct validity of the SCIDS-C was established by factorial validity and concurrent validity. Internal consistency and test-retest reliability was demonstrated in this study. In short, acceptable validity and reliability were established.

The 4-factor structure of the original version was supported in the SCIDS-C by the results of the CFA, except that one of the model fit indices, RMSEA, was slightly higher than the criterion. To facilitate international comparisons, we keep the same factor structure in the SCIDS-C. Correlations between the SCIDS-C and the DKAS and between the SCIDS-C and the ADQ were positive but weak, indicating that the three instruments were measuring similar yet distinct concepts. This echoed with the definition of sense of competence in dementia care, which refers to caregivers’ self-perceived competence in providing dementia care on the basis of knowledge, attitudes and skills [22]. Positive correlation was also found between the total scores of the English version of SCIDS and ADQ [23] and that with the subscale score of ‘Person-centred’ of ADQ (r = 0.13) [22]. However, correlation between the English version of SCIDS and other instruments for assessing dementia knowledge, such as Dementia Knowledge Twenty [22, 48] and Dementia Knowledge Assessment Tool Version Two [23], was not reported. This may be attributed to the different nature of the instruments. Dementia Knowledge Twenty has marginal consistency reliability (Cronbach’s α value of .63 and .67) and Dementia Knowledge Assessment Tool Version Two is for family carers focusing on late stages of dementia and has poor convergent validity and ceiling effects [49].

The known-groups comparison of the SCIDS-C scores between care assistants and health professionals was partly demonstrated because only two subscale scores were significantly different. This observation is inconsistent with the findings of a recent study conducted in Hong Kong which showed that significantly differences were found in the total scores of the SCIDS among different types of staff, including care assistants, supporting staff, professionals, and managerial staff [50]. Such comparison has not been conducted in the studies conducted in the UK or Japan [22, 31]. It is noteworthy that the care assistants scored higher than the health professionals in subscale ‘Professionalism’. This may be because health professionals in nursing homes and hospitals are mainly responsible for the medical care and are less involved in personal care for people with dementia [51]. More works are needed to explore the differences and similarities in their perceived competencies between the two groups as this would provide insights into intervention development for equipping care staff with knowledge as well as appropriate attitude and skills [52] .

Similar to the English version [22] and the Japanese version [31], good internal consistency was demonstrated for the SCIDS-C. However, the Cronbach’s α of the three subscales were slightly lower than the criterion, which probably because there was only few items in each scale [46].

The sense of competence among the respondents in this study was lower than the nursing home staff in Australia (average score of 49.95) [23], but better than the counterparts in Hong Kong (average score of 49.95) [50]. The major gaps in their sense of competence were revealed by the items with the lowest scores as three out of four are within the subscale ‘Building Relationships’. It appears that the respondents lack confidence in their skills for communicating and understanding people with dementia and meeting their care needs. These concerns echoed with the findings of previous studies in the mainland China. For example, many healthcare providers could not differentiate symptoms of dementia from those of mental disorders [19]. Nursing home staff misbelieved that residents with dementia are uncommunicative and thus were not willing to talk to them [51] and could not understand their care needs [53]. Education for healthcare providers on the relevant pathophysiology and communication skills is pertinent for capacity building in dementia care in China.

### Limitations

We acknowledged two study limitations and so the findings should be interpreted with caution. Firstly, the respondents were recruited from metropolitan areas, whose background and available medical or training resources are generally better than those in rural areas [54]. The results of the level of sense of competence might not represent all healthcare providers in mainland China. Secondly, the experience of dementia care training was not collected. This factor was not being taken into account in examining known-group validity.

### Implication

Healthcare providers’ sense of competence in dementia care is essential for meeting the complex care needs of people with dementia. The SCIDS-C can be a measure to assess gaps in knowledge, attitudes and skills for dementia care, and thereby revealing the educational needs of healthcare providers in the Chinese communities. Further study can identify factors contributing to the perceived sense of competence and examine the sensitivity of the SCIDS-C to detect change in sense of competence in dementia care across time.

## Conclusion

In summary, the SCIDS was translated into Chinese by following a rigorous translation process. The validation study also demonstrated satisfactory validity and reliability in terms of content validity, concurrent validity, internal consistency and test-retest reliability, among Chinese healthcare providers. The CFA model of the 4-factor structure was marginally fit in current study because the RMSEA was slightly higher than the criterion. The known-groups validity between professional and care assistants was partially established. The findings of SCIDS-C score also indicates the educational gap related to the healthcare provides’ dementia-care competence.

Healthcare providers’ sense of competence in dementia care is fundamental for meeting the complex needs of people with dementia, thereby providing quality care for them. This study provides a validated instrument for assessing the Chinese healthcare providers’ level of sense of competence in dementia care, which will provide insight into their needs in equipping with dementia-care competence. The SCIDS-C can be a reliable instrument for evaluating the effects of relevant educational intervention.

## Supplementary Information

**Additional file 1.** STROBE (Strengthening The Reporting of OBservational Studies in Epidemiology) Checklist.

**Additional file 2.** Mean score and frequency of responses to each item in the Chinese version of Sense of Competence in Dementia Care Staff scale.

## Data Availability

All data generated or analysed during this study are included in this published article and its supplementary file 2. The original datasets are available from the corresponding author on reasonable request.

## Abbreviations

SCIDS: Sense of Competence in Dementia Care Staff
SCIDS-C: Chinese version of the Sense of Competence in Dementia Care Staff
DKAS: Dementia knowledge assessment scale
ADQ: Approaches to Dementia Questionnaire
I-CVI: Content validity index of each item
S-CVI: Scale-level of content validity index
CFA: Confirmatory factor analysis
CFI: Comparative fit index
RMSEA: Root mean squared error of approximation
SRMR: Standardized root mean square residual
ICC: Intra-class correlation coefficient
