# Supplementary material for: Translation and validation of Chinese version of sense of competence in dementia care staff scale in healthcare providers: a cross-sectional study
Source: BMC Nurs. 2022 Jan 30;21:35. doi: 10.1186/s12912-022-00815-3 (PMC8801082; doi:10.1186/s12912-022-00815-3)
Supplement: Supplementary file 2 — Additional file 2. Mean score and frequency of responses to each item in the Chinese version of Sense of Competence in Dementia Care Staff scale. [file 12912_2022_815_MOESM2_ESM.docx]

# **Supplementary file 2** Mean score and frequency of responses to each item in the Chinese version of Sense of Competence in Dementia Care Staff scale

| How well do you feel you can… | Not at all | A little bit | Quite a lot | Very much | Mean | SD |
| --- | --- | --- | --- | --- | --- | --- |
|  | *n* (%) | *n* (%) | *n* (%) | *n* (%) |  |  |
| 1. Understand the feelings of a person with dementia? | 15(5.17) | 110(37.93) | 133 (45.86) | 31 (10.69) | 2.62 | 0.75 |
| 2. Understand the way a person with dementia interacts with the people and things around them? | 26 (8.97) | 92 (31.72) | 151 (52.07) | 19 (6.55) | 2.57 | 0.75 |
| 3. Engage a person with dementia in a conversation? | 13 (4.48) | 120 (41.38) | 134 (46.21) | 21 (7.24) | 2.57 | 0.70 |
| 4. Balance the needs of the person with dementia with their relative’s wishes and the service’s limitations? | 25 (8.62) | 110 (37.93) | 129 (44.48) | 23 (7.93) | 2.52 | 0.77 |
| 5. Use information about their past (such as what they used to do and their interests), when talking to a person with dementia? | 10 (3.45) | 72 (24.83) | 159 (54.83) | 48 (16.55) | 2.85 | 0.73 |
| 6. Change your work to match the changing needs of a person with dementia? | 14 (4.83) | 100 (34.48) | 141 (48.62) | 33 (11.38) | 2.67 | 0.74 |
| 7. Keep up a positive attitude towards the people you care for? | 7 (2.41) | 44 (15.17) | 177 (61.03) | 61 (21.03) | 3.01 | 0.68 |
| 8. Keep up a positive attitude towards the relatives of a person with dementia? | 7 (2.41) | 46 (15.86) | 166 (57.24) | 69 (23.79) | 3.03 | 0.71 |
| 9. Keep yourself motivated during a working day? | 5 (1.72) | 60 (20.69) | 164 (56.55) | 60 (20.69) | 2.97 | 0.70 |
| 10. Play an active role in your staff team? | 4 (1.38) | 83 (28.62) | 155 (53.45) | 47 (16.21) | 2.85 | 0.70 |
| 11. Protect the dignity of a person with dementia in your work? | 5 (1.72) | 36 (12.41) | 173 (59.66) | 75 (25.86) | 3.10 | 0.67 |
| 12. Deal with personal care, such as incontinence in a person with dementia? | 12 (4.14) | 63 (21.72) | 143 (49.31) | 71 (24.48) | 2.94 | 0.79 |
| 13. Deal with behavior that challenges in a person with dementia? | 7 (2.41) | 118 (40.69) | 129 (44.48) | 34 (11.72) | 2.66 | 0.71 |
| 14. Decide what to do about risk (such as harm to self or others) in a person with dementia? | 12 (4.14) | 112 (38.62) | 142 (48.97) | 22 (7.59) | 2.60 | 0.70 |
| 15. Offer stimulation (for the mind, the senses and the body) to a person with dementia in your daily work? | 22 (7.59) | 115 (39.66) | 130 (44.83) | 21 (7.24) | 2.52 | 0.74 |
| 16. Offer choice to a person with dementia in everyday care (such as what to wear, or what to do)? | 7 (2.41) | 79 (27.24) | 167 (57.59) | 34 (11.72) | 2.79 | 0.67 |
| 17. Engage a person with dementia in creative activities during your normal working day? | 14 (4.83) | 106 (36.55) | 133 (45.86) | 35 (12.07) | 2.66 | 0.75 |

*Note.* Scoring: All items are scored from 1 (Not at all) to 4 (Very much). Higher scores mean a higher level of sense of confidence.

Scores are added up for items from 1 to 17 for the overall SCIDS score (ranging from 17 to 68) and for the subscales as follows:

Professionalism: 7, 8, 9, 10, 12 (scores ranging from 5 to 20); Building Relationships: 1, 2, 3, 4 (scores ranging from 4 to 12);

Care Challenges: 13, 14, 15, 17 (scores ranging from 4 to 12); and Sustaining Personhood: 5, 6, 11, 16 (scores ranging from 4 to 12).

(Reference: Schepers, A. K., Orrell, M., Shanahan, N., & Spector, A. (2012). Sense of Competence in Dementia Care Staff (SCIDS) scale: Development, reliability, and validity. *International Psychogeriatrics*, *24*(7), 1153–1162. https://doi.org/10.1017/S104161021100247X)
